# Induced Pluripotent Stem Cell-Differentiated Chondrocytes Repair Cartilage Defect in a Rabbit Osteoarthritis Model

**DOI:** 10.1155/2020/8867349

**Published:** 2020-11-09

**Authors:** Yu-Hsun Chang, Kun-Chi Wu, Dah-Ching Ding

**Affiliations:** ^1^Department of Pediatrics, Hualien Tzu Chi Hospital, Buddhist Tzu Chi Medical Foundation, and Tzu Chi University, Hualien, Taiwan; ^2^Department of Orthopedics, Hualien Tzu Chi Hospital, Buddhist Tzu Chi Medical Foundation, and Tzu Chi University, Hualien, Taiwan; ^3^Department of Obstetrics and Gynecology, Hualien Tzu Chi Hospital, Buddhist Tzu Chi Medical Foundation, and Tzu Chi University, Hualien, Taiwan; ^4^Department of Research, Hualien Tzu Chi Hospital, Buddhist Tzu Chi Medical Foundation, and Tzu Chi University, Hualien, Taiwan; ^5^Institute of Medical Sciences, Tzu Chi University, Hualien, Taiwan

## Abstract

The aim of this study was to explore the therapeutic effect of iPSC-mesenchymal stem cell (MSC)-derived chondrocytes in a rabbit osteoarthritis (OA) model. The iPSCs were characterized by gene expressions, immunostaining of pluripotent markers, and *in vivo* teratoma formation. iPSC-differentiated MSCs were characterized by flow cytometry and trilineage differentiation. A rabbit OA model was established by the transection of the anterior cruciate ligament. The therapeutic effect of transplanted iPSC-MSC-chondrocytes on the OA was evaluated by the histology, immunostaining, and qPCR of defective cartilage. The results showed iPSC could express pluripotency markers such as OCT4, SOX2, and NANOG and form an embryoid body and a teratoma. After differentiation of iPSCs for 30 days, MSCs were established. The iPSC-MSC could express typical MSC markers such as CD29, CD44, CD90, CD105, and HLA-ABC. They could differentiate into adipocytes, osteocytes, and chondrocytes. In this model, iPSC-MSC-chondrocytes significantly improved the histology and ICRS (International Cartilage Repair Society) scores. The transplanted cartilage expressed less IL-1*β*, TNF-*α*, and MMP13 than control cartilage. In conclusion, the iPSCs we derived might represent an emerging source for differentiated MSC-chondrocyte and might rescue cartilage defects through its anti-inflammatory and anti-catabolic effects.

## 1. Introduction

Osteoarthritis (OA) is characterized by a gradual articular cartilage loss due to unknown causes [1]. Chondrocytes would produce proteolytic enzymes, including metalloproteinases and aggrecanases, to destroy collagen and proteoglycans [2]; gradually loss of cartilage may lead to joint space narrowing and underlying bone exposure [3]. OA has an impact on the health of both men and women worldwide and about 10%–15% of adults over 60 years old have some degree of OA. It is becoming an increasingly important disease, ranked as a cause for second disability [4].

The current symptoms' control treatments of OA include both pharmacological and nonpharmacological approaches [5] as well as knee joint replacement in cases of severe cartilage destruction [6]. However, these treatment modalities still lack a definite efficacy. Regeneration therapies, including autologous serum injection, platelet-rich plasma, and mesenchymal stem cell (MSC) transplantation, have been proposed [7–10]. Mechanisms of such therapies are anti-inflammation and chondrocyte regeneration by MSCs [11, 12]. However, cell source, number of derived cells, and invasive harvesting procedures represent a limitation to their use [13].

Characteristics of induced pluripotent stem cells (iPSCs) are the same as embryonic stem cells. After introducing Oct4, sox2, Nanog, c-myc, and klf4 genes, the cells can turn back to the embryonic stage and have pluripotent characteristics [14, 15]. Their use for therapeutic purposes has also emerged, such as for retinal regeneration [16]. In addition, iPSCs have been found to differentiate into chondrocytes via mesenchymal progenitors with embryoid body formation [17–19]. However, the use of progenitor cells, which may be contaminated with pluripotent stem cells, has caused tumor generation [20]. Therefore, the elimination of pluripotency cell contamination is critical for successful iPSC therapy [21].

As chondrocytes represent a cell type with terminal differentiation, the iPSC-differentiated chondrocytes might be associated with a decreased chance of tumor formation after transplantation. The other drawbacks of iPSC included instability of the induced phenotype and possible accelerated senescence [22]. Several ways to conquer accelerated senescence are in combination with the SV40 large T antigen (SV40 LT) and/or hTERT [23] or knockdown p53 expression [24].

The aim of the present study was to use iPSC-differentiated MSC-derived chondrocytes to repair cartilage defects with OA and explore the therapeutic mechanism.

## 2. Materials and Methods

### 2.1. The Culture of iPSCs

The iPSC line was developed from normal human epidermal keratinocytes (NHEK) cell line bought from PromoCell (Heidelberg, Germany). The whole study protocol was followed by the institutional guidelines and approved by the Research Ethics Committee, Hualien Tzu Chi Hospital (IRB 104-46-A).

iPSC cells were generated using Sendai virus reprogramming [25]. Briefly, the NHEK cell line was maintained in culture plates precoated with EpiLife medium (Invitrogen, Carlsbad, CA, USA) and supplemented with EpiLife human keratinocytes growth supplement (HKGS) (Invitrogen) at 37°C in a 5% CO2 incubator. We changed the medium every 48 hours and subcultured them when the cells reached 75% of confluence. We used a nonintegrating system to introduce four genes (CytoTune™-iPS 2.0 Sendai reprogramming kit [Invitrogen, Carlsbad, CA, USA, Cat. No. A16517]). The system used Sendai virus particles to deliver polycistronic KLF4–OCT3/4–SOX2, CMYC, and KLF4. NHEK cells were seeded at a density of 5 × 10^4^ cells per well (34.8 mm in diameter) in EpiLife medium. We changed the medium every other day until the cells reached a confluence of 40% with small clusters formation at 2-3 days. Then, we transduced the Sendai virus vector at an MOI of 4 : 4 : 2 (KLF4–OCT3/4–SOX2:CMYC: KLF4) in the medium. After 24 hours, the cells were washed with 1X Dulbecco's PBS (-Ca2+/Mg2+) (Gibco, Massachusetts, United States) and fed with fresh EpiLife medium containing HKGS. At 7 days posttransfection, we passaged cells onto vitronectin (rhVTN-N) (Gibco, Massachusetts, United States)-coated 6-well plates at a density of 2 × 10^4^ cells/well. We fed every day with chemically defined Essential 8 medium (Gibco, Massachusetts, United States) and 10 *μ*M Y-27632 (Merck Millipore Massachusetts, United States). After 12 days of transduction, we manually isolated positive TRA-1-60 or TRA-1-81 colonies under live cell staining. We passaged the iPSC with the EDTA method (0.5 M EDTA, pH 8.0, Invitrogen, Carlsbad, CA, USA) [26].

The resulted iPSCs were cultured on MEF feeder cells [27] with a culture media which consisted of 80% (*v*/*v*) knockout (KO) DMEM, 20% (*v*/*v*) KO serum replacement, 2 mM L-glutamine, 10 mM nonessential amino acid (Invitrogen, Waltham, MA, USA), and 4 ng/mL basic fibroblast growth factor (bFGF).

### 2.2. Pluripotency of iPSC

iPSCs' pluripotency was evaluated by immunostaining and RT-PCR for Oct4, Sox2, and Nanog (pluripotency markers). *In vitro* differentiation was demonstrated with markers of three germ layers [brachyury (mesoderm), Tuj-1 (ectoderm), and AFP (endoderm)] by means of embryoid body formation, for 5 days. In order to evaluate the iPSCs' pluripotency phenotype, we performed *in vivo* differentiation. Specifically, iPSCs were subcutaneously injected in nonobese diabetes and severe combined immunodeficient (NOD-SCID) mice. After 3 months, the xenograft tumors were examined. The three germ layers' differentiation was demonstrated by RT-PCR. The latter included endoderm (GATA4), mesoderm (Hand1 and GATA6), and ectoderm (Tubb3, MAP2, and GFAP).

### 2.3. Protocol for Differentiation into iPSC-Derived MSC Cells

We modified a previously published protocol to differentiate iPSC into MSC [28]. We dissociated undifferentiated iPSC with 4 mg/ml of collagenase I to remove MEFs and any differentiated cells. The resulting iPSC colonies were dissociated by manual pipetting and then placed onto Matrigel-coated dishes. Subsequently, iPSC media was changed to the MSC culture medium. The MSC culture medium contained DMEM-low glucose (DMEM-LG, Gibco, Grand Island, NY, USA) with 10% fetal bovine serum (FBS, Biological Industry, Kibbutz, Israel), 100 units/mL of Penicillin and Streptomycin (Gibco). The MSC medium was changed every day for a total of 5 days. Cells were then detached from the Matrigel-coated dishes using 2.5% trypsin/0.23 mM ethylenediaminetetraacetic acid (EDTA, Sigma) at 37°C for 10 min. Cells were subsequently cultured on uncoated culture dishes. The medium was changed every 3 days. When reaching a 90% confluence of 7 days of culture, cells were passaged with a 1 : 3 ratio. The morphology of iPSCs and iPSC-MSCs was observed by a microscope image (Nikon, Tokyo, Japan). This step took nearly one month.

### 2.4. Flow Cytometry of iPSC–MSC Cells

MSC-specific surface markers were evaluated and characterized by flow cytometry. iPSC-derived MSCs were detached using 2 mM EDTA in PBS and washed. They were then incubated with the candidate antibody conjugated with phycoerythrin (PE) or fluorescein isothiocyanate (FITC). Specifically, candidate antibodies included the following: CD14, CD19, CD24, CD29, CD34, CD44, CD45, CD56, CD73, CD90, CD105, CD117, CD133, HLA-ABC, and HLA-DR (BD, Pharmingen). Becton Dickinson flow cytometer (Vantage SE, Becton Dickinson, San Jose, CA, USA) was used for the analysis.

### 2.5. *In vitro* Adipogenic and Osteogenic Differentiation of iPSC-MSCs

To evaluate the iPSC-derived MSCs' *in vitro*adipogenic and osteogenic potential, cells were detached from the dish and underwent progeny differentiation. Adipogenic differentiation was induced by culturing 3 × 10^5^ cells/cm^2^ in an adipogenic medium. The latter consisted of DMEM supplemented with 10% FBS, 1 mmol/L dexamethasone, 5 mg/mL insulin, 0.5 mmol/L isobutylmethylxanthine, and 60 mmol/L indomethacin (Sigma). The medium was changed every 3 days for 3 weeks. Osteogenic differentiation was induced by culturing 6 × 10^4^ cells/cm^2^ in the osteogenic differentiation medium. Such a medium consisted of DMEM supplemented with 10% FBS, 0.1 mmol/L dexamethasone, 10 mmol/L *β*-glycerol phosphate, and 50 mmol/L ascorbate (Sigma). Adipogenic differentiation was evaluated based on the presence of lipid droplets in the cells through 0.3% Oil Red O stain (Sigma) for 15 min at room temperature. Osteogenic differentiation was evaluated by mineralization of calcium accumulation by Alizarin Red S staining (Sigma).

### 2.6. *In vitro* Chondrogenic Differentiation of iPSC–MSC

To study the chondrogenic potential of iPSC-derived MSCs, cells were detached from the dish, and chondrogenic differentiation took place in a micromass method [25]. Briefly, 1 × 10^5^ iPSC-derived MSCs were grown in the chondrogenic medium. The latter consisted of DMEM, 10% FBS, 10 ng/mL transforming growth factor-*β*1 (TGF-*β*1), 50 *μ*g/mL of ascorbic acid-2-phosphate, and 6.25 *μ*g/mL of insulin. The medium was changed every 3 days. The chondrogenic medium was changed every other day for 21 days. Micromasses were formed after differentiation for 21 days and sent for histology, immunostaining, and gene expression analysis.

### 2.7. *In vivo* Tumor Xenografts

iPSCs were detached by mechanical cutting, and the resulting sheets were resuspended in Matrigel with PBS (BD, 1 : 1). The iPSCs obtained were kept on ice for less than 60 min in order to maintain viability. The cells were then injected subcutaneously into the back tissue of NOD-SCID mice (*n* = 3). Tumor formation follow-up was performed by palpation up to 3 months. Xenograft tumors were evaluated by gene expressions of three germ layer differentiation, *GATA4* (endoderm); *HAND1* and *GATA6* (mesoderm); and *TUBB3*, *MAP2*, and *GFAP* (ectoderm) and compared to their expressions of iPSCs.

### 2.8. RT-PCR

In order to clarify the gene expression of pluripotency and differentiation in iPSC, RT-PCR was performed. We also detected the gene expressions of potassium channels in iPSC-MSC and BMSC. In order to extract total ribonucleic acid (RNA) from iPSC-MSCs, BMSC, the micromass (*n* = 3) obtained from the cell cultures or tumor tissue from xenografts (*n* = 3), we used an RNeasy Protect Mini Kit with an efficient on-column RNase-free DNase treatment (Qiagen, Hilden, Germany). RNA was eluted in 30 mL of RNase-free water. RT-PCR was performed using a SuperScript III One-Step RT-PCR kit (Invitrogen, Grand Island, NY, USA) according to the manufacturer's instructions.

### 2.9. Quantitative Real-Time PCR (qPCR)

To confirm the progeny differentiation of the iPSC-derived MSCs, qPCR was utilized. Gene expression of the chondrogenic differentiated cells was assessed for hyaline cartilage markers [i.e., type 2 collagen (*COL2A1*) and Aggrecan (*ACAN*)].

The glyceraldehyde 3-phosphate dehydrogenase (*GAPDH*) was used as an internal control. For quantification, we used the expression of the following genes: *FABP4* and *PPARγ* for adipogenesis, and osteopontin (*OPN*) and alkaline phosphatase (*ALPL*) for osteogenesis. For inflammatory markers of transplanted cartilage, rabbit *IL-1β*, *TNF-α*, and *MMP13* were tested. Rabbit type 2 collagen *(COL2A)* and type 1 collagen (*COL1*) were also tested for hyaline cartilage formation. Gene expression changes from iPSC to MSC were tested for *OCT4* (a pluripotency gene) and *CD73* (a marker of MSC).  Table 1 lists the primer sequences used. As to the qPCR procedures, in brief, real-time PCRs were performed and monitored using FastStart Universal SYBR Green Master (ROX, Roche, Indianapolis, IN, USA) and a qPCR detection system (ABI Step One Plus system, Applied Biosystems, Foster City, CA, USA). Each target gene's expression levels were calculated as 2^-*ΔΔ*Ct^ [29]. Three readings for each experimental sample were obtained for each gene of interest. Additionally, the experiments were repeated in triplicates.

### 2.10. Immunohistochemistry

In order to investigate cells' production of aggrecan and type 2 collagen, immunostaining with type 2 collagen and aggrecan was performed. Chondrogenesis derived micromasses were fixed in 10% formalin (Sigma), then washed with graded alcohol (70%, 95%, and 100%) to dehydrate and to make tissue transparent with xylene (EMD, Millipore, USA). Micromasses were subsequently embedded in paraffin, sectioned with 4 *μ*m thickness, and mounted on glass slides. Hematoxylin and Eosin (H&E) staining was performed to evaluate cell morphology. The Safranin O staining was used to evaluate glycosaminoglycan levels. Finally, immunostaining for type 2 collagen and aggrecan was performed for the detection of related protein expression. Specifically, for immunohistochemistry, tissue sections were predigested with pepsin (1 mg/mL in Tris-HCl, pH 2.0) and incubated with type 2 collagen and aggrecan primary antibody (1 : 200, EMD; Millipore) for 60 min. Reactivity was detected by a diaminobenzidine tetrahydrochloride substrate. We stained cartilage tissue with an anti-human mitochondria antibody (1 : 200, Sigma-Aldrich) to detect whether human cells were integrating and regenerating after 12 weeks of transplantation. A mouse anti-rabbit CD45 antibody (1 : 200, Bio-Rad, Hercules, CA, USA) was used for detecting rabbit CD45 cells located in cartilage. A digital camera under a light microscope (Nikon, Tokyo, Japan) was used to capture images of the stained sections.

### 2.11. Animal Experiments

In the present study, we used three 14-month-old New Zealand White rabbits. First, the rabbits' knees received an anterior cruciate ligament transection (ACLT) as previously described procedure [30]. Then, they were divided into right knees, OA + 1.8 × 10^8^ iPSC–MSC-chondrocytes, and left knees, OA with 0.5 mL of saline (*n* = 3).

### 2.12. Surgical Procedure of OA Rabbit Model

The Animal Studies Ethics Committee of Tzu Chi University approved the experiment's protocol (105-26-2). We confirmed that all methods were performed in accordance with the relevant guidelines and regulations. Adult (48–60 months old, 3.3–4.6 kg) male New Zealand white rabbits (*n* = 3) were anesthetized by an intramuscular injection of xylazine (8 mg/kg) and ketamine (100 mg/kg). Both knees were shaved and disinfected with a beta-iodine solution. A medial parapatellar incision through the skin was made, and an arthrotomy was performed. The patella was dislocated laterally, and the knee was placed in a full flexion manner. A no. 15 blade was used to transect the anterior cruciate ligament (ACL). The joint was then irrigated with sterile saline and closed with a running suture of 3-0 vicryl (Ethicon, Johnson & Johnson, Bridgewater, NJ, USA). Subsequently, a mattress suture of 3-0 nylon (Ethicon) was used to close the skin. All rabbits were returned to their cages after the surgery and allowed to freely move. For pain relief, rabbits received 0.2 mg/kg of oral meloxicam daily for 7 days. The rabbits after the procedures were well in feeding and living on the following days. Two adult (48–60 months old, 3.3–4.6 kg) male New Zealand white rabbits without any procedure were used as normal control.

### 2.13. Treatment of iPSC–MSC-Chondrocytes in OA Rabbit Model

After 8 weeks from ACLT, the right knee joints of the three rabbits were injected with 0.5 mL 0.9% normal saline (Otsuka, Taipei, Taiwan) mixed with 1.8 × 10^8^ iPSC–MSC-chondrocytes (1 × 10^6^ iPSC-MSCs were cultured in a 15 cm dish with the supplement of the chondrocyte differentiation medium stated for above 3 weeks). As an internal control, at the same time, the left knee joints were injected with 0.5 mL saline. Subsequently, 12 weeks after cell therapy, we used pentobarbital (3%) 15–40 mg/kg to sedate the rabbits and sacrificed them by CO_2_ inhalation.

### 2.14. Tissue Harvest

Posteuthanasia, joint surfaces were grossly evaluated. The proximal, tibial, and distal femoral plateaus were removed. Specimens were decalcified with Decalcifier II (Leica Surgipath, Harbourfront Centre, Singapore) for 3–5 days, then placed into 10% formalin, and cut into four pieces. All four pieces were paraffin-embedded. We performed serial sagittal sections, 4 *μ*m in thickness.

### 2.15. Histological Evaluation

Tissue sections were stained with H&E (Sigma) to evaluate cellular architecture and 0.1% Safranin O solution (Sigma) to detect cartilage repair and to demonstrate the matrix's proteoglycan contents. Immunohistochemical staining was performed on additional sections. Specifically, an aggrecan antibody (1 : 200), anti-human nuclei antibody (1 : 200), and anti-rabbit CD45 (1 : 200) were used for immunohistochemistry. Reactivity was detected with a diaminobenzidine tetrahydrochloride substrate. Images were captured with a digital camera under a light microscope (Nikon, Tokyo, Japan).

Quantification of aggrecan+ cells in cartilage was performed using 3 fields near injury sites and number of aggrecan+ cells among 50 cells counted in each field.

Quantitative evaluation of cartilage repair was performed using cartilage thickness and using the International Cartilage Repair Society (ICRS) scoring system [31]. We randomly selected three slides of the three rabbits and counted the thickness of the destruction site with or without iPSC-MSC-chondrocytes transplantation. Subsequently, the mean and standard deviation of the cartilage's thickness was calculated. With the ICRS score, we scored the cartilage from the three rabbits with six categories including the surface, matrix, cell distribution, cell population viability, subchondral bone, and cartilage mineralization. The scores ranged from 0 to 18.

### 2.16. Statistical Analysis

All data were expressed as mean and standard deviation. Statistical comparisons of the gene expressions, cartilage's thickness, aggrecan+ cells, and ICRS scores in both groups were performed with nonparametric tests, such as the Mann–Whitney *U* test. To compare three genes' expression levels, we used ANOVA test with a post hoc analysis with the Bonferroni test. A *p* value < 0.05 was considered as statistically significant. All statistical analyses were performed on SPSS (version 22 for Windows; IBM, Inc., New York, NY, USA).

## 3. Results

### 3.1. Characterization of iPSCs and the Derived Embryoid Body and Xenograft Tumor

**Figure 1:**
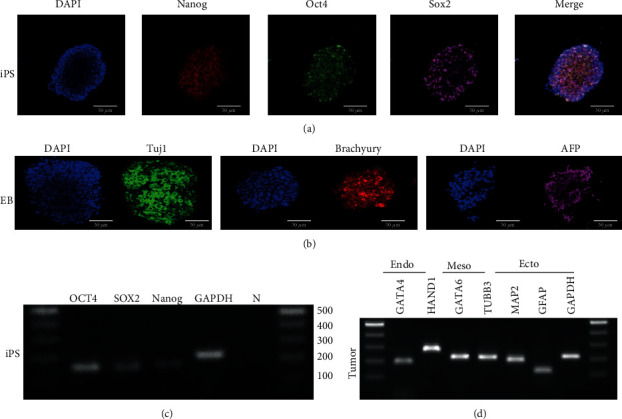
Characterization of iPSCs, embryoid body, and teratoma. (a) Counterstaining the nucleus with DAPI as blue color. Immunofluorescence staining against pluripotency markers including NANOG (red), OCT4 (green), and SOX2 (pink). The last figure was a merged picture. Scalebars = 50*μ*m. (b) Counterstaining the nucleus with DAPI as blue color. Immunofluorescence staining of embryoid body differentiation against TUJ-1 (ectoderm, green in color), BRACHYURY (mesoderm, red in color), and AFP (endoderm, pink in color). (c) RT-PCR of pluripotency gene expressions of iPSCs. The iPSCs were positive for pluripotency markers including *OCT4*, *SOX2*, and *NANOG*. (d) RT-PCR of gene expression of iPSC-derived teratoma. The teratoma expressed genes of three germ layers, including *GATA4* (endoderm), *HAND1*, *GATA6* (mesoderm), *TUBB3*, *MAP2*, and *GFAP* (ectoderm).

**Figure 2:**
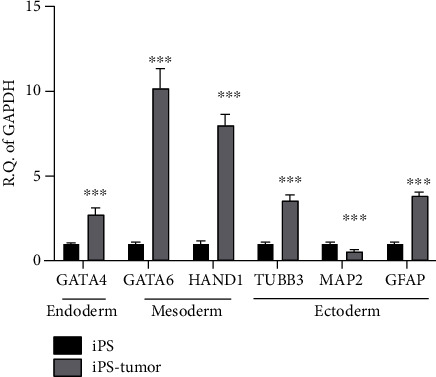
Gene expressions of iPSC-derived teratoma compared to iPSC. qPCR showed teratoma expressing more gene expressions of *GATA4* (endoderm), *HAND1*, *GATA6* (mesoderm), *TUBB3*, *MAP2*, and *GFAP* (ectoderm) than iPSC. Statistical analyses between both groups were calculated with Mann–Whitney *U* test. ^∗∗∗^*p* < 0.001.

The pluripotency of iPSCs was evaluated by demonstrating that iPSCs immunofluorescence staining against NANOG, OCT4, and SOX2 were positive (Figure 1(a)). Of note, immunofluorescence staining of the *in vitro* differentiated embryoid body showed positive against three germ layer markers including brachyury (mesoderm), tuj-1 (ectoderm), and AFP (endoderm) (Figure 1(b)). RT-PCR evaluation of the pluripotent gene expression of iPSCs demonstrated that they could express *NANOG*, *OCT4*, and *SOX2* (Figure 1(c)). Additionally, RT-PCR also indicated the gene expression of iPSC-derived tumor tissue in three germ layers, demonstrating expressions of *GATA4* in endoderm; *HAND1* and *GATA6* in mesoderm; and *TUBB3*, *MAP2*, and *GFAP* in ectoderm (Figure 1(d)). These germ layers gene expressions except for MAP2 were more increased in xenograft than in the iPSCs (Figure 2). Taken together, iPSCs were pluripotent and could differentiate into three germ layers *in vitro* and *in vivo*.

### 3.2. The Process of MSCs Generation from iPSCs

**Figure 3:**
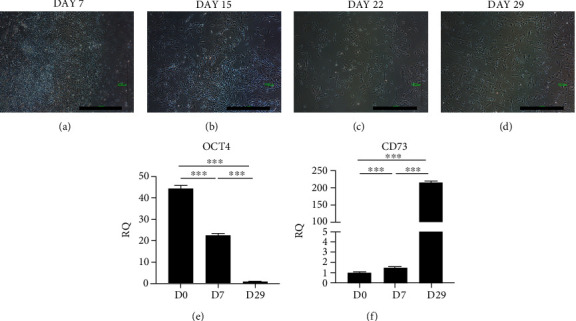
The process from iPSC to mesenchymal stem cell (MSC). (a) The fibroblastic morphology cells (Lt side of the figure) appeared on day 7 after differentiation around the epithelial round cell colony (Rt side of the figure). (b) At day 14, more fibroblast-like cells appeared. (c) At day 21, around 100% of cells were fibroblastic appearance cells. (d) On day 29, fully differentiated MSC were noted. Scalebar = 1000*μ*m. (e) qPCR showed a decreasing trend of pluripotency gene (*OCT4*) expression from postdifferentiation day 7 to day 29. (f) qPCR showed an increasing trend of MSC gene (*CD73*) expression from postdifferentiation day 7 to day 29. ^∗∗∗^*p* < 0.001. We used an ANOVA test with a post hoc analysis with the Bonferroni test.

Undifferentiated iPSCs were plated onto a Matrigel-coating dish on Day 0. Subsequently, the medium was changed to the MSC culture medium (DMEM +10% FBS), once every 3 days. On Day 7 (Figure 3(a)), spindle-shaped cells appeared around the colony core (left side of the figure). At one week later, these cells were passaged to an uncoated dish for subsequently enriched cultures (Day 15, Figure 3(b)). When the cells reached 70% confluence, they were passaged at a ratio of 1 : 3. On Days 22 (Figure 3(c)) and 29 (Figure 3(d)), most of the cell population was spindle-shaped. Figures 3(e) and 3(f) show that gene expression changed from iPSCs to MSCs, indicating the *OCT4* was less expressed and *CD73* more expressed in MSCs than in iPSCs. Taken together, MSC was differentiated successfully from iPSC after 30 days of differentiation.

### 3.3. Characterization of iPSC-Derived MSCs

#### 3.3.1. Surface Markers

**Figure 4:**
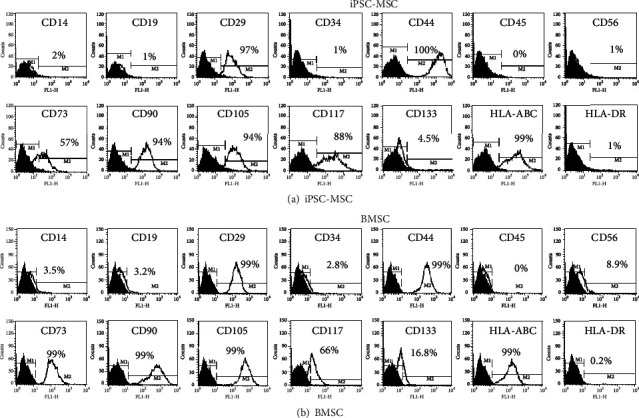
Flow cytometry of iPSC-MSC and bone marrow stem cells (BMSC). (a) Surface marker expression of iPSC-derived MSCs. They have positive expressions of CD29, CD44, CD73, CD90, CD105, CD117, and HLA-ABC and negative for CD14, CD19, CD34, CD45, CD56, CD133, and HLA-DR. (b) Surface markers expression of BMSC. The expression patterns were similar to iPSC-MSC.

After 30 days of MSCs differentiation process, the surface markers of iPSC-derived MSCs by flow cytometry were positive for CD29, CD44, CD73, CD90, CD105, CD117, and HLA-ABC and negative for CD14, CD19, CD34, CD45, CD56, CD133, and HLA-DR (Figure 4(a)). The surface markers panel was almost the same as bone marrow stem cells (BMSCs) (Figure 4(b)). In summary, iPSC-MSC surface marker expressions were as similar as BMSC.

### 3.4. The Expression of CD24, CD105, and Potassium Channel of iPSC-MSC and BMSC

**Figure 5:**
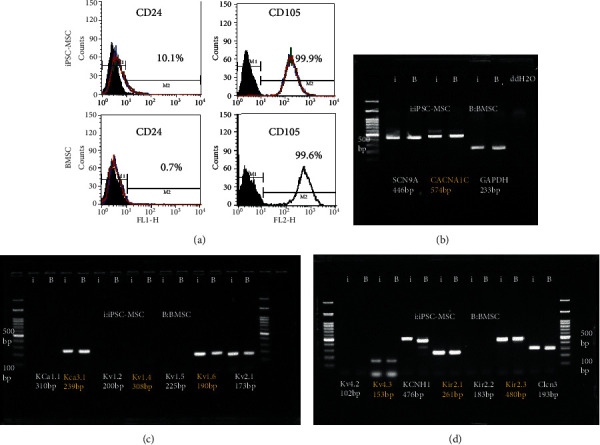
CD24, CD105, and potassium channel expression in iPSC-MSC and BMSC. (a) CD24 and CD105 expressions in iPSC-MSC and BMSC. CD24 was expressed differently between both cells (10% in iPSC-MSC and 0.7% in BMSC). (b–d) RT-PCR showing the potassium channel expressions in iPSC-MSC and BMSC. Both cells expressed potassium channel genes similarly.

Regarding the purity of differentiated iPSC-MSC, previous study has shown isolated CD24(-) CD105(+) iPSC-MSC could generate pure MSC and MSC could express different potassium channel patterns [32–35]. Therefore, we compared the CD24, CD105, and potassium channel in both cells. We found that there were 10% iPSC-MSC expressed CD24 and 99.9% iPSC-MSC expressed CD105, whereas 0.7% BMSC expressed CD24 and 99.6% BMSC expressed CD105 (Figure 5(a)). Potassium channel expressions were the same between iPSC-MSC and BMSC (Figures 5(b)–5(d)). Taken together, the expression pattern of iPSC-MSC was almost the same as BMSC except CD24.

### 3.5. Differentiation of iPSC-Derived MSCs

**Figure 6:**
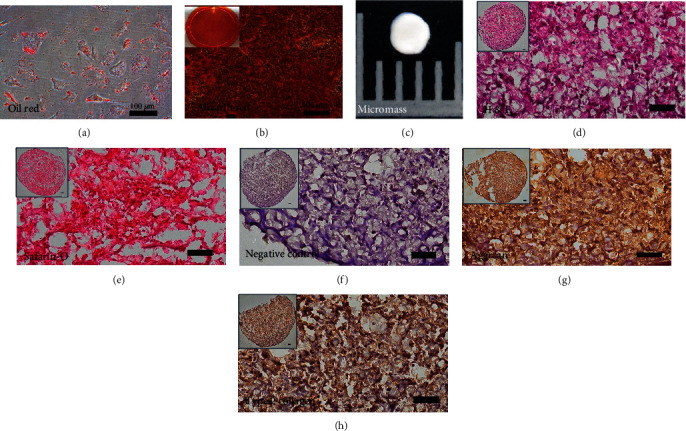
Trilineage differentiation of iPSC-derived MSCs. (a) After the adipogenesis of iPSC–MSC for 2 weeks, the oil droplets in the cells were staining positive for Oil red. Scalebar = 100*μ*m. (b) After the osteogenesis of iPSC–MSC for 2 weeks, the mineral deposition in cells was staining positive for Alizarin Red. Insert picture was Alizarin Red staining of one well of a 6-well plate. Scalebar = 100*μ*m. (c) After chondrogenesis of iPSC-derived MSCs for 21 days, the iPSC–MSC formed a cartilage micromass. The picture is 5 mm in length. (d) The Hematoxylin and Eosin (H & E) staining of the micromass. (e, g, h) Micromasses were positive for sulfated glycosaminoglycans (Safranin O) (e), aggrecan (g), and type 2 collagen (h). (f) Negative control staining for immunohistochemistry (without the first antibody). Insert pictures in (d–h) were the picture of the whole micromass section. Scalebar = 100*μ*m.

Next, we checked the differentiation capability of iPSC-derived MSCs. In the adipogenesis of iPSC–MSC, the cells showed positive staining for Oil red, an adipose cell marker (Figure 6(a)). In osteogenesis of iPSC–MSCs, the cells were positive for Alizarin Red, an osteocyte marker (Figure 6(b)). Following chondrogenesis of iPSC-derived MSCs for 3 weeks, the cells formed a micromass (Figure 6(c)). H&E staining of the micromass showed a cartilage-like appearance (Figure 6(d)). The micromass was positive for Safranin O (sulfated glycosaminoglycans, Figure 6(e)), aggrecan (Figure 6(g)), and type 2 collagen (Figure 6(h)) staining.  Figure 6(f) presents negative control of immunohistochemistry.

**Figure 7:**
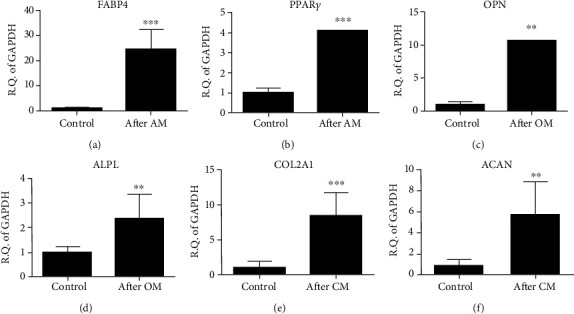
Gene expressions of iPSC–MSC trilineage differentiations. (a–f) qPCR showed an increased expression of (a, b) adipocyte-related genes (*FABP4* and *PPARγ*), (c, d) osteocyte-related genes (osteopontin [*OPN*] and alkaline phosphatase [*ALPL*]), and (e, f) chondrocyte-related genes (type II collagen [*COL2A1*] and aggrecan [*ACAN*]) genes postdifferentiation. AM: adipogenesis medium; OM: osteogenesis medium; CM: chondrogenesis medium. ^∗∗^*p* < 0.01, ^∗∗∗^*p* < 0.001. The experiment was performed thrice. Statistical analyses between both groups were calculated with Mann–Whitney *U* test.

Quantitative PCR was used to demonstrate the gene expressions of trilineage differentiation of iPSC–MSC. It showed an increased expression of adipocyte-related genes, *FABP4* and *PPARγ* (Figures 7(a) and 7(b)); osteoblast-related genes, osteopontin [*OPN*] and alkaline phosphatase [*ALPL*] (Figures 7(c) and 7(d)); and chondrocyte-related genes, *type II collagen* and *aggrecan* (Figures 7(e) and 7(f)) after differentiation. Taken together, iPSC-MSC could proceed trilineage differentiation.

### 3.6. Microscopic Findings after *in vivo* Transplantation

**Figure 8:**
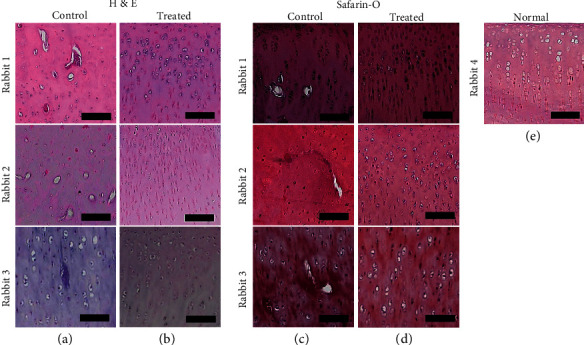
The histology of cartilages with or without iPSC–MSC-chondrocytes transplantation (*n* = 3). (a, b) The H & E staining of histology of the cartilage of the osteoarthritis (OA) rabbit model without (a) and with cell transplantation (b). (c, d) Safranin O staining of cartilage without (c) or with (d) cells transplantation. (e) Normal rabbit cartilage with Safranin O staining. After iPSC–MSC-chondrocytes transplantation, the morphology and sulfated glycosaminoglycan expressions of transplanted cartilages were more similar to normal cartilage than control cartilage. Scalebar = 100*μ*m.

**Figure 9:**
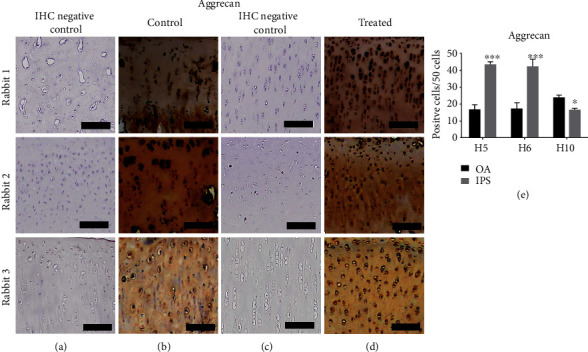
The immunohistochemistry (IHC) in iPSC–MSC-chondrocytes transplant cartilages (*n* = 3). (a, b) The IHC of aggrecan in control cartilage with IHC-negative control (a) and staining cartilage (b). (c, d) IHC of aggrecan in treated cartilage with IHC-negative control (c) and staining cartilage (d). Scalebar = 100*μ*m. (e) Quantification of aggrecan+ cells in 50 cells. More aggrecan+ cells/50 cells were noted in rabbit 1 and 2. ^∗^*p* < 0.5, ^∗∗∗^*p* < 0.01. Statistical analyses between both groups were calculated with Mann–Whitney *U* test.

After ACLT 8 weeks, we transplanted iPSC-differentiated MSC-derived chondrocytes (iPS-MSC-chondrocytes) to repair cartilage defects with OA. After 12 weeks of transplantation, slices of cartilage were evaluated by histology and immunohistochemistry staining. The iPSC-MSC-chondrocytes transplanted cartilage demonstrated more intact histology (Figures 8(a) and 8(b)), positivity for Safranin O (Figures 8(c) and 8(d)), and aggrecan (Figure 9). Figure 9(e) shows the quantification of aggrecan+ cells per 50 cells in the cartilage. There were more aggrecan+ cells noted in the transplanted cartilage.

**Figure 10:**
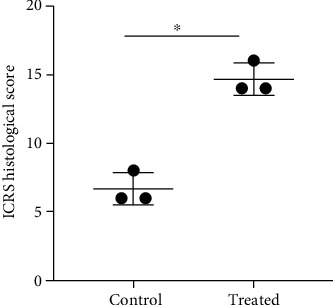
The International Cartilage Repair Society (ICRS) histological score for evaluating tissue regeneration in osteochondral defects in rabbit articular cartilage at 12 weeks posttreatment. The ICRS histological scores in the treatment group (iPSC–MSC-chondrocytes) were significantly higher than those in the control group (*n* = 3). ^∗^*p* = 0.02. Statistical analyses between both groups were calculated with Mann–Whitney *U* test.

In a semiquantitative analysis of the sections, the histological assessment of ICRS scores of the three rabbits showed that the repaired tissues in the treated knees were histologically superior to those in the control knees (Figure 10, *n* = 3, *p* = 0.02).

Taken together, microscopically, we proved iPSC-MSC-chondrocytes transplanted cartilages were more intact than control cartilage.

### 3.7. iPSC-MSC-Chondrocytes Transplantation Decreased Inflammatory Cytokines and Catabolic Matrix Protein Expression

**Figure 11:**
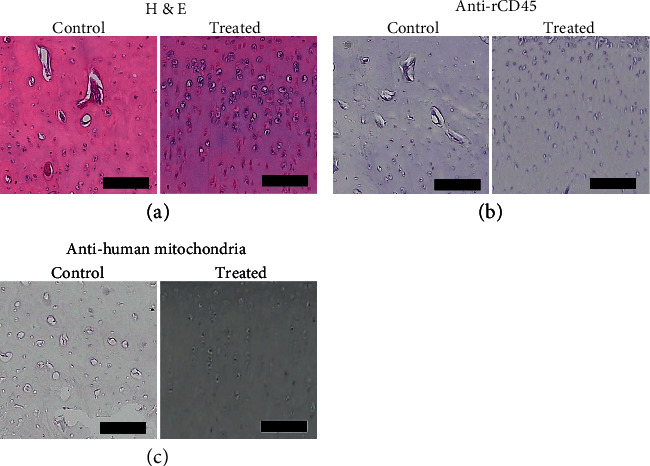
The histology and immunohistochemistry of the iPSC-MSC-chondrocytes transplanted cartilage. (a) H & E staining of control and treated cartilage. The control cartilage was more destructive than treated cartilage. (b) The IHC of rCD45. There was no staining of rCD45 in both groups. (c) Anti-human mitochondria staining of the cartilage with or without transplanted iPSC-MSC-chondrocytes. There was no staining of anti-human mitochondria in treated cartilage. Scalebar = 100*μ*m.

To know the mechanism of how the cartilage defect was repaired by iPSC-MSC-chondrocytes, we performed IHC staining with an antibody against human mitochondria and rabbit CD45, and gene expressions of several inflammatory markers and type 1 and 2 collagen. In H & E staining, we found control cartilage showed more destruction of cartilage and less chondrocytes than the treated (Figure 11(a)). There were no rabbit CD45 cells activation in both control and treated cartilage (Figure 11(b)). We did not find human cells integrated into the rabbit cartilage (Figure 11(c)).

**Figure 12:**
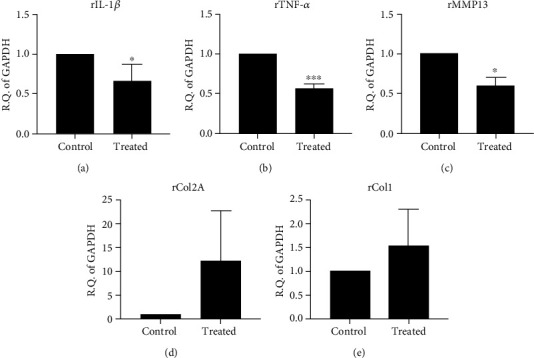
The qRT-PCR of inflammation, catabolic, and cartilage matrix gene expressions in the cartilage with or without transplanted chondrocytes (*n* = 3). (a, b) The inflammatory cytokines, *rIL-1β* (a) and *rTNF-α* (b), were illustrated. The inflammatory cytokines decreased expression after cell transplantation. (c) Catabolic matrix gene expression of *rMMP13*. The expression of the catabolic gene decreased expression after cell transplantation. (d) Gene expression of cartilage matrix of type 2 collagen [*rCOL2A*]. The expression of type 2 collagen was not different between control and treated groups. (e) Gene expression of fibrocartilage marker (type 1 collagen [*rCOL1*]). The expression of type 1 collagen was not different between control and treated groups. ^∗^*p* < 0.05, ^∗∗∗^*p* < 0.001. Statistical analyses between both groups were calculated with Mann–Whitney *U* test.

We found inflammatory and catabolic markers, including IL-1*β*, TNF-*α*, and MMP13, decreased expressions after iPS-MSC-chondrocytes transplant (Figures 12(a)–12(c)). However, we did not find the difference of type 2 collagen expression between control and transplanted cartilage (Figure 12(d)). The type 1 collagen expression was also not significantly different between both groups (Figure 12(e)).

Taken together, the mechanism of iPSC-MSC-chondrocytes might contribute to their anti-inflammatory and anticatabolic characteristics.

## 4. Discussion

In the present study, we demonstrated that iPSCs can differentiate into MSCs after 30 days, without an embryoid body formation process. The MSCs could successfully differentiate into chondrocytes within 3 weeks. Of note, we found that the differentiated chondrocytes effectively repaired cartilage defects *in vivo* by anti-inflammatory and anticatabolic mechanisms.

iPSCs offer several advantages over bone marrow-derived stem cells (BMSCs) and chondrocytes for cartilage repair [36]. Specifically, iPSC can provide an abundant cell source and a patient-specific *in vitro* model to study genetic and environmental factors influencing the pathogenesis of OA. Moreover, BMSC and adipose stem cells revealed limited differentiation capability after passage 4 [37]. On the contrary, iPSC can provide an indefinite expansion [38]. The other cell source with an indefinite expansion ability is the embryonic stem cells (ESCs). However, the ESCs harbor allogeneic properties, generating immune reactions posttransplantation, limiting their clinical application potential [38]. Compared with other MSC sources, the generation process is minimally invasive, such as taking skin fibroblasts or blood cells, even from OA suffering elderly population [39].

The iPSC owned a comparable immunomodulation effect than MSC. Previous studies demonstrated that MSCs have immunosuppressive effects on mitogen-activated immune cells [40, 41]. The iPSC-MSC is also proved to inhibit mitogen-activated peripheral blood mononuclear cells (PBMC) [42]. The iPSC-MSC owns low tumorigenicity and strong immunomodulatory effects [43]. Moreover, iPSC-MSC could induce immune tolerance after transplantation to support long-term survival of graft [44]. Taken together, iPSC-MSC might be a good alternative cell source.

Although iPSC has numerous advantages in cell number, expansion, and differentiation capabilities, the use of iPSC for cartilage repair has been restricted to date, due to the complexity of differentiation protocol and the inefficiency of chondrocytes generation. Currently, four methods are known for the generation of iPSC-derived chondrocytes [45]. The first one is monolayer culture with a defined growth factor [46]; the second one is differentiation into the embryoid body with chondrogenic medium, with TGF-*β*3 cells [47]; the third one is a conditioned medium derived from chondrocytes and added in the culture medium of the embryoid body; and the last one is embryoid body cultured in a chondrocyte condition medium-plus growth factor of TGF-*β*3. Of note, MSCs selected from the embryoid body are costly and laborious, providing low cellular yields [48, 49]. It was concluded that monolayer culture with growth factors and culture with chondrocyte condition medium were the most effective methods [45]. Our study used monolayer culture in which iPSCs differentiated firstly into MSCs, and then differentiated into chondrocytes. This approach was more efficient in chondrogenesis from iPSC than from EB [46].

Other differentiation methods omitted the formation of the embryoid body, including micromass formation of iPSC [50], pellet condition [51], and cultures on a modified dish or feeder layers [52]. However, these methods may have problems such as difficult clinical translation due to the following reasons: influence on cell proliferation by the substrate properties, decrease of related signaling pathways, mixture with feeders, and increased cost [46]. In an additional study, a different method used a TGF-*β* inhibitor to suppress the relative signal in order to enhance MSC conversion [53]. Nevertheless, to date, an in-between tread of the level of epithelialization needed and TGF-*β* inhibitor's safety have not been established. In the present study, we used the easiest way of iPSC-derived chondrocyte via monolayer culture, adding growth factors to facilitate chondrocyte production.

OCT4 expression is abundant in iPSC and reveals the characteristic of pluripotency [54]. Our study showed the iPSC marker and transcription factor OCT4 expression in iPSCs was almost completely lost in the cells after 29 days of transition of iPSCs toward MSCs. The CD73 is one of the classic markers of MSCs [55]. We also demonstrated CD73 was dramatically (200-fold) induced after iPSCs transition toward MSCs.

Regarding purity of iPSC-MSC, previous studies showed they isolated iPSC-MSC CD24(-) CD105(+) cells to generate MSC population [32–34]. These cells could propagate more than 100 passages and perform trilineage differentiations. Besides, human iPSC-MSCs express potassium channel Kir2.2 and Kir2.3 but BMSC do not [35]. Nevertheless, in our study, we found the iPSC-MSC was CD24 low expression (10%) and CD105 (+). The potassium channel expressions between both kinds of cells were the same, such as Kir2.2 and Kir2.3. Therefore, the purity of iPSC-MSC may be proved.

Prolonged cell culture may induce chondrocytes undergoing hypertrophic changes (i.e., expression of the type X collagen gene (COL10A1) [56]. In addition, the lack of specific markers as chondrocyte identity makes it difficult to prospectively isolate chondrocytes. Recent fate-mapping studies in the mouse show that chondrocyte-like cells are derived from early notochord-like nucleus pulposus cells (NP cells) [57]. The efforts to derive notochord-like NP cells for chondrocyte differentiation have been also reported recently [58]. The advantages of NP cell-derived chondrocytes are that NP cells are the proceeding cells of the chondrocytes and could be easily identified. The disadvantages of NP cell-derived chondrocytes are needed for one more step of differentiation than iPSC-MSC.

OA is caused by a chronic inflammatory process. Chondrolysis may be caused by increased inflammatory cytokines such as IL-1*β*, TNF-*α*, and catabolic matrix protein MMP13 in the joint [59]. In addition, IL-1*β* could also inhibit the production of cartilage matrix components such as type 2 collagen and aggrecan [60]. It might be possible that the anti-inflammation effects were from iPSC-MSCs instead of iPSC-MSC-chondrocytes. Recent studies also indicated mitochondrial transfer of iPSC-MSC is another putative mechanism to reduce inflammation and change metabolic status of injured cells/tissues [61–63]. In our study, we found that transplanted iPSC-MSC-chondrocytes could inhibit these inflammatory cytokines and catabolic matrix protein.

During iPSC differentiation to MSC (iPSC-MSC), gene expressions of characteristic surface markers of MSC, including CD44, CD73, and CD105, were upregulated [64]. However, the immune modulation function of iPSC-MSC was not as well as genuine MSC [64]. The previous study showed that iPSC-MSC showed rejuvenation from methylation associated with age and senescence compared to genuine MSC [64]. It means iPSC-MSC owns differentiation and immunomodulation advantages of MSC but not owns age- and senescence-associated disadvantages. In our study, we could see the surface marker expressions and differentiation capability of iPSC-MSC were the same as genuine MSC.

The mechanisms of stem cells repair the destroyed cartilage are contributed by stem cell integration and differentiation, immunomodulation, or by the paracrine factors and exosome production [65]. One previous study showed human iPSC, transplanted or cotransplanted with alginate into defected cartilage, could be integrated into defected cartilage and repair [66]. The other study revealed that MSC owned immunomodulation and anti-inflammatory effects to repair the cartilage defect [67]. One review also pointed out that autologous chondrocyte implantation (ACI) is the most established clinical treatment currently [68]. However, there are several disadvantages of ACI including the complexity and cost of surgical procedures, the biological response of the flap, and the complexity of the in vitro expansion of chondrocytes [69]. In our study, we could provide several advantages over ACI. Firstly, the cell numbers are unlimited for iPSC, and the procedures of culturing fibroblast and iPSC are not complex. Secondly, we found chondrocytes differentiated from iPSC-MSC could repair the defective cartilage through the anti-inflammation effect. The chondrocytes would not integrate into defective cartilage but decreased several inflammatory markers such as IL-1*β*, TNF-*α*, and MMP13. To our knowledge, these findings were not reported before. Thirdly, the methylation status of iPSC-MSC was different from the MSC. The iPSC-MSC is rejuvenated.

Some limitations can be identified in the present study. First, FBS was used as a nutrient in the culture medium. This could increase the hazard of cell transformation and prion contamination [70]. In the future, for clinical application, a serum replacement could decrease the safety concern [71]. Second, iPSC could undergo teratoma formation. However, here, we performed a terminal differentiation, like chondrocyte differentiation. Therefore, there was a very small chance of teratoma formation. Of note, we did not observe teratoma formation in our experiments. Furthermore, we used the Sendai virus to transfect four factors. The virus is expected to eliminate after 10–13 passages in culture, decreasing the chances of pluripotency factors' reactivation [72]. We did not see the iPSC-MSC-chondrocytes integration after 12 weeks of transplantation. The cells may die after such a long period of time. A short time period of the experiment to see if iPSC-MSC-chondrocytes integration is needed in the future.

## 5. Conclusion

In conclusion, the present study shows the iPSCs we derived could directly differentiate into MSCs and subsequently into chondrocytes, in the absence of embryoid body formation. These iPSC-MSC-chondrocytes might effectively repair cartilage defects through anti-inflammatory and anticatabolic mechanisms. Thus, the iPSCs we derived might represent a feasible source of cell therapy for OA-damaged cartilage.

## Figures and Tables

**Table 1:** Gene primer sequences used for RT-PCR and qPCR experiments.

Gene name	Forward sequence	Reverse sequence	Base pair no.
*Oct4*	CAG TGC CCG AAA CCC ACA C	CAG TGC CCG AAA CCC ACA C	161
*Nanog*	AGT CCC AAA GGC AAA CAA CCC ACT TC	TGC TGG AGG CTG AGG TAT TTC TGT CTC	161
*Sox2*	GGG AAA TGG GAG GGG TGC AAA AGA GG	TTG CGT GAG TGT GGA TGG GAT TGG TG	151
*MAP2*	GCA TGA GCT CTT GGC AGG	CCA ATT GAA CCC ATG TAA AGC C	194
*GFAP*	AGG GCT GAC ACG TCC AC	GCC TTA GAG GGG AGA GGA G	132
*GATA4*	TCC CTC TTC CCT CCT CAA AT	TCA GCG TGT AAA GGC ATC TG	194
*Tubb3*	CAG AGC AAG AAC AGC AGC TAC TT	GTG AAC TCC ATC TCG TCC ATG CCC TC	227
*Hand1*	TGC CTG AGA AAG AGA ACC AG	ATG GCA GGA TGA ACA AAC AC	274
*GATA6*	CCT CAC TCC ACT CGT GTC TGC	GTC CTG GCT TCT GGA AGT GG	225
*PPARr*	AGC CTC ATG AAG AGC CTT CCA	TCC GGA AGA AAC CCT TGC A	120
*OPN*	AGG AGG AGG CAG AGC ACA	CTG GTA TGG CAC AGG TGA TG	150
*APAL*	CCACGTCTTCACATTTGGTG	GCAGTGAAGGGCTTCTTGTC	96
*FABP4*	ATGGGATGGAAAATCAACCA	GTGGAAGTGACGCCTTTCAT	87
*Aggrecan*	CGAAACATCACTGAGGGTGA	GCAAACGTGAAGGGCTCCT	107
*Col2A1*	GAGAGGTCTTCCTGGCAAAG	AAGTCCCTGGAAGCCAGAT	118
*CD73*	AGTCCACTGGAGAGTTCCTGCA	TGAGAGGGTCATAACTGGGCAC	133
*OCT4*	CTT GCT GCA GAA GTG GGT GGA GGA	CTG CAG TGT GGG TTT CGG GCA	169
*GAPDH*	GGTCTCCTCTGACTTGAACA	GTGAGGGTCTCTCTCTTCCT	221
*KCa1.1*	ACAACATCTCCCCCAACC	TCATCACCTTCTTTCCAATTC	310
*KCa3.1*	CGGGAACAAGTGAACTCCAT	ACTGGGGAAAGTAGCCTGGT	239
*Kv1.2*	ATGAGAGAATTGGGCCTCCT	CCCACTATCTTTCCCCCAAT	200
*Kv1.4*	ACGAGGGCTTTGTGAGAGAA	CACGATGAAGAAGGGGTCAT	308
*Kv1.5*	GTAACGTCAAGGCCAAGAGC	GGGAGGAAAGGAGTGAAAGG	225
*Kv1.6*	CTGGCTTGACCACAGTCTGA	CTGGAGTTTGCCTGAGGAAG	190
*Kv2.1*	GTTGGCCATTCTGCCATACT	GCAAAGTGAAGCCCAGAGAC	173
*Kv4.2*	GCTTGTCATCAATCCCCTTG	TCCAGTATCTGGGCTTTTCC	102
*Kv4.3*	ACGGAGACATGGTGCCTAAG	CCCTGCGTTTATCAGCTCTC	153
*KCNH1*	TGGATTTTGCAAGCTGTCTG	GAGTCTTTGGTGCCTCTTGC	476
*Kir2.1*	AACAGGGAGGTGTGGACAAG	TAACCTGCTCTAGGGCTCCA	261
*Kir2.2*	GAGGCTATCACAGGCTCAGG	CCCCAAGTTAAAAACCAGCA	183
*Kir2.3*	GCTTTGAGCCTGTGGTCTTC	TTGGCTCTGTCCTGAGTGTG	480
*Clcn3*	CATAGGTCAAGCAGAGGGTC	TATTTCCGCAGCAACAGG	293
*SCN9A*	GCTCCGAGTCTTCAAGTTGG	GGTTGTTTGCATCAGGGTCT	446
*CACNA1C*	AACATCAACAACGCCAACAA	AGGGCAGGACTGTCTTCTGA	574
*GAPDH*	CCATCTTCCAGGAGCGAG	GCAGGAGGCATTGCTGAT	233
*rCollagen 2a*	CCTGTGCGACGACATAATCTGT	GGTCCTTTAGGTCCTACGATATCCT	176
*rCOL1*	CAGAACGGCCTCAGGTACCA	CAGATCACGTCATCGCACAAC	101
*rTNFa*	CTGCACTTCAGGGTGATCG	CTACGTGGGCTAGAGGCTTG	133
*rIL1B*	TTGAAGAAGAACCCGTCCTCTG	CTCATACGTGCCAGACAACACC	128
*rMMP13*	AGGAAGACCTCCAGTTTGCAGAG	GCTGCATTCTCCTTCAGGATTC	85
*rGAPDH*	TGACGACATCAAGAAGGTGGTG	GAAGGTGGAGGAGTGGGTGTC	120

## Data Availability

The original data can ask the corresponding author.

## Abbreviations

ACL: Anterior cruciate ligament
ACLT: Anterior cruciate ligament transection
FBS: Fetal bovine serum
H&E: Hematoxylin and Eosin
MSC: Mesenchymal stem cell
PRP: Platelet-rich plasma.
